# Payments by Patients

**Published:** 1920-05-22

**Authors:** 


### Payments by Patients.

### What the Hampstead General Hospital Proposes.

The Council of the Hampstead General and North-West London Hospital has decided to ask all patients able to do so to contribute towards the hospital expenses while receiving treatment. Experience shows that there are few who cannot afford to give something, and a suggestion in this direction almost invariably meets with ready response. At the present time a large number of out-patients contribute sixpence a week towards the cost of medicines. This alone produces not less than £8 a week. practice is being extended to general anæsthetics administered in the out-patient department, for which many are pleased to pay 2s. 6d. The payment of 1s. for dental anæsthetics has been customary for many years. Cases requiring radiographic examination, involving considerable expenditure on plates, have hitherto been overlooked, but there is little doubt that many will be glad to pay 2s. 6d., the contribution suggested. A payment of 6d. to 1s. per attendance suggested for massage and electrical treatment should be well within the means of the greater number who attend this department. While it is difficult to estimate the annual sum which will be collected in this manner, it will obviously help considerably in defraying the heavy expenses involved. It has been customary for many years to write to all patients after their discharge asking them when in a position to do so to remember the needs of the Institution which has served them. The only change it is proposed to make is to send a circular letter to the patient prior to admission or, in the case of emergencies, to the patients' relatives immediately after admission, indicating the weekly cost of maintenance, and asking for a promise to pay such a proportion of the cost as might be feasible. A slip and addressed envelope are enclosed for the purpose of securing their promise.

